# Investigating the Prognostic and Oncogenic Roles of Membrane-Associated Ring-CH-Type Finger 9 in Colorectal Cancer

**DOI:** 10.1155/2024/9279653

**Published:** 2024-08-17

**Authors:** Jiayan Zhang, Qinghan Jiao, Zhigang Chen

**Affiliations:** ^1^ Department of Gynecology The Second Affiliated Hospital and Yuying Children's Hospital of Wenzhou Medical University, Wenzhou, Zhejiang, China; ^2^ Department of Nuclear Medicine The Second Affiliated Hospital and Yuying Children's Hospital of Wenzhou Medical University, Wenzhou, Zhejiang, China; ^3^ Department of Interventional The Second Affiliated Hospital and Yuying Children's Hospital of Wenzhou Medical University, Wenzhou, Zhejiang, China

## Abstract

*Backgroundsand Aims*. Colorectal cancer (CRC) represents a major global health challenge, necessitating comprehensive investigations into its underlying molecular mechanisms to enhance diagnostic and therapeutic strategies. This study focuses on elucidating the oncogenic role of Membrane-Associated Ring-CH-Type Finger 9 (MARCHF9), a RING-Type E3 ubiquitin transferase, in CRC. We aim to assess MARCHF9's clinical significance, functional impact on CRC progression, and its potential as a prognostic biomarker. *Methods*. We leveraged data from the Cancer Genome Atlas (TCGA) cohort to evaluate MARCHF9 expression profiles in CRC. In vitro experiments involved siRNA-mediated MARCHF9 knockdown in COAD cell lines (SW480 and LoVo). Cell proliferation and invasion assays were conducted to investigate MARCHF9's functional relevance. Survival analyses were performed to assess its prognostic role. *Results*. Our analysis revealed significantly elevated MARCHF9 expression in CRC tissues compared to normal colorectal tissues (*P* < 0.05). High MARCHF9 expression correlated with advanced clinical stages, distant metastases, and the presence of residual tumors in CRC patients. Survival analyses demonstrated that high MARCHF9 expression predicted unfavorable overall and disease-free survival outcomes (*P* < 0.05). In vitro experiments further supported its oncogenic potential, with MARCHF9 knockdown inhibiting COAD cell proliferation and invasion. *Conclusions*. This study unveils the oncogenic role of MARCHF9 in CRC, highlighting its clinical relevance as a potential biomarker and therapeutic target. MARCHF9's association with adverse clinicopathological features and its functional impact on cancer cell behavior underscore its significance in CRC progression. Further research is essential to elucidate precise mechanisms by which MARCHF9 enhances tumorigenesis and to explore its therapeutic potential in CRC management.

## 1. Introduction

Colorectal cancer (CRC) is a major global health concern and a leading cause of cancer-related deaths, accounting for over 1.8 million new cases and approximately 880,000 deaths worldwide in 2018 alone, according to the World Cancer Research Fund International [1]. This malignancy is characterized by the uncontrolled growth of cells in the colon or rectum, with a multifaceted etiology that includes genetic, environmental, and lifestyle factors [2]. Despite significant advances in the understanding of CRC pathogenesis and treatment modalities, there remains a pressing need to unravel the intricate molecular mechanisms underlying this disease [3].

In recent years, the E3 ubiquitin ligases have emerged as critical regulators of cancer development and progression, offering new insights into potential therapeutic targets [4]. These enzymes, which mediate the attachment of ubiquitin moieties to target proteins, play a pivotal role in the regulation of protein stability, intracellular trafficking, and signaling pathways [5]. MARCHF9 (Membrane-Associated Ring-CH-Type Finger 9), a RING-Type E3 ubiquitin transferase, has gained attention due to its implications in diverse physiological processes, including immune regulation, cellular signaling, and protein turnover [6–8]. While our understanding of MARCHF9's functions in normal physiology has expanded, its role in carcinogenesis, particularly in colorectal cancer, remains poorly elucidated.

The purpose of this study is to investigate the oncogenic role of MARCHF9 in colorectal cancer. Understanding the molecular mechanisms by which MARCHF9 may contribute to CRC development and progression holds significant promise for the development of novel diagnostic markers and targeted therapies. In this paper, we provide a comprehensive overview of colorectal cancer and highlight the significance of MARCHF9 in cellular processes. Through this investigation, we hope to contribute valuable insights to the field of cancer biology and potentially pave the way for more effective treatment strategies for colorectal cancer patients.

## 2. Methods

### 2.1. Online Dataset

The online dataset for this study was obtained from the Cancer Genome Atlas (TCGA), a comprehensive resource that provides genomic and clinical data for various cancer types, including colorectal adenocarcinoma (COAD) and rectum adenocarcinoma (READ). We also enrolled data from the Gene Expression Omnibus (GEO) datasets.

### 2.2. Cell Culture

The human colorectal adenocarcinoma cell lines SW480 and LoVo were obtained from ATCC. Cells were maintained in DMEM for SW480 and RPMI 1640 for LoVo, supplemented with 10% fetal bovine serum (FBS) and 1% penicillin-streptomycin (pen-strep). Cells were cultured at 37°C in a humidified atmosphere containing 5% CO2 [9].

### 2.3. Cell Transfection by siRNA

To investigate the functional role of MARCHF9, small interfering RNA (siRNA) transfection was performed. siRNAs targeting MARCHF9 and negative control siRNAs (si-MARCH9: 5″-GCAGTGGAAGGTCCTAAATTA-3″, si-control: 5″-UUCUCCGAACGUGUCACGU-3″) were transfected into COAD cells using Lipo3000 Transfection reagent. The validated siRNA has been shown to effectively reduce MARCHF9 expression, minimizing off-target effects [10], which supports its use in our experiments. The transfection efficiency was validated by subsequent western blot experiments.

### 2.4. Western Blotting

After siRNA transfection, total protein was extracted from COAD cells using RIPA lysis buffer, supplemented with protease and phosphatase inhibitors. Then protein concentrations were determined using a BCA protein assay kit (Pearson). Protein samples were separated by sodium dodecyl sulfate-polyacrylamide gel electrophoresis (SDS-PAGE), transferred onto polyvinylidene difluoride (PVDF) membranes, and blocked with 5% non-fat milk in Tris-buffered saline with Tween 20 (TBST). Membranes were probed with primary antibodies against MARCHF9 (PA5-103817; Thermo Fisher Scientific, PA, USA) and GAPDH (loading control) and subsequently incubated with appropriate secondary antibodies. Protein bands were visualized using an enhanced chemiluminescence (ECL) detection system, and images were captured and semiquantified [11].

### 2.5. MTT Assay

Cell proliferation was assessed using the 3-(4,5-dimethylthiazol-2-yl)-2,5-diphenyltetrazolium bromide (MTT) assay [12]. Following MARCHF9 siRNA transfection, COAD cells were seeded in 96-well plates, and MTT solution was added to each well according to manufacturer's procedures. Absorbance measurements were recorded at appropriate time points to evaluate cell viability (OD 570 nm at 24, 48, 72, and 96 hours).

### 2.6. Matrigel-Transwell Assay

To assess cell invasion, transfected COAD cells were subjected to Matrigel-Transwell assays [13]. Cells were seeded in the upper chamber of Transwell inserts coated with Matrigel (356234, Corning, NY, USA). The lower chamber contained culture medium with 10% FBS as a chemoattractant. After incubation, non-invading cells were removed from the upper surface of the membrane, while invading cells on the lower surface were fixed, stained, and quantified using microscopy.

### 2.7. Survival Analyses and Statistics

Survival analyses were conducted to assess the prognostic role of MARCHF9 in COAD patients. Kaplan–Meier survival curves were established for overall survival and disease-specific survival. Log-rank test was applied to evaluate significance of survival differences. Cox hazard regression test was conducted to identify independent risk factors. Data from in vitro experiments were analyzed using Student's *t*-test. The significance level was set at *P* < 0.05. Statistical analyses were performed using R software, SPSS 24.0 software, and GraphPad Prism 7.0 Software.

### 2.8. Ethics

This study involving human cell lines did not require ethical approval as it does not involve human subjects or patient data. Cell lines used in this study were obtained from established cell repository (ATCC) and maintained according to standard laboratory protocols. All experiments were conducted in compliance with relevant ethical guidelines and biosafety regulations.

## 3. Results

### 3.1. Expression Difference of MARCHF9 in Colorectal Cancers and Pan-Cancers

**Figure 1:**
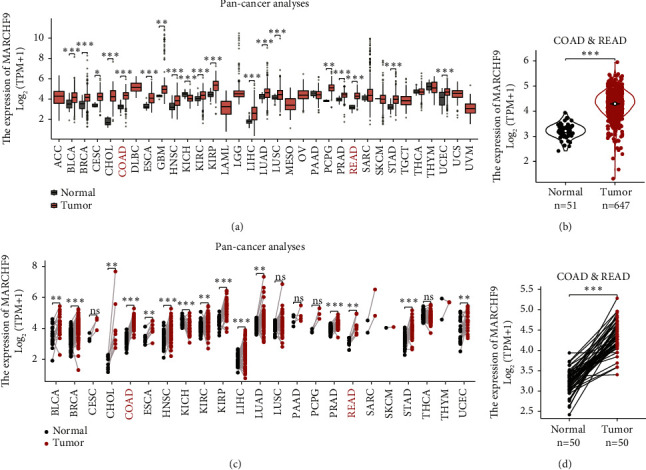
Expression difference of MARCHF9 in colorectal cancers and pan-cancers. (a) Differential mRNA levels of MARCHF9 between pan-cancer tissues and corresponding normal tissues. Analysis conducted using unpaired Student's *t*-test based on the Cancer Genome Atlas (TCGA) dataset. (b) Distinct mRNA levels of MARCHF9 in COAD and READ compared to normal colorectal tissues. Analysis performed using unpaired Student's *t*-test based on TCGA dataset. (c) Variations in mRNA levels of MARCHF9 in pan-cancers compared to their respective paired normal tissues. Analysis conducted using paired Student's *t*-test based on TCGA dataset. (d) Differential mRNA levels of MARCHF9 in COAD and READ relative to their paired normal colorectal tissues. Analysis performed using paired Student's *t*-test based on TCGA dataset. ^*∗*^*P* < 0.05.

To gain insights into the potential role of MARCHF9 in CRC and various other cancer types, we conducted a comprehensive analysis of MARCHF9 expression using data from the Cancer Genome Atlas (TCGA) cohort. Initially, we observed significant variations in MARCHF9 mRNA levels in pan-cancer tissues compared to normal tissues. This analysis, performed using an unpaired Student's *t*-test, revealed notable upregulation of MARCHF9 expression in multiple cancer types, including bladder and breast cancer (Figure 1(a)). Consistent with these findings, CRC (comprising COAD and READ) also exhibited a marked increase in MARCHF9 expression compared to normal tissues (Figure 1(b)). These results highlight the potential significance of MARCHF9 dysregulation in colorectal cancer.

To further refine our analysis, we performed a paired analysis to compare MARCHF9 expression in cancer tissues with their corresponding paired normal tissues. Across pan-cancer samples, this analysis revealed a consistent upregulation of MARCHF9 expression in cancer tissues, emphasizing its potential as a candidate oncogene (Figure 1(c)). Similarly, when focusing on COAD and READ samples, the paired analysis highlighted a significant increase in MARCHF9 expression in cancer tissues relative to paired normal colorectal tissues (Figure 1(d)). These findings, determined using a paired Student's *t*-test, reinforce the notion that MARCHF9 may play a pivotal role in the development and progression of colorectal cancer.

### 3.2. Clinical Characteristics of Colorectal Cancer Patients Stratified by MARCHF9 Expression

Our study next delved into the clinical implications of MARCHF9 expression in CRC by stratifying patients into low and high MARCHF9 expression groups (Table 1). While gender and age distributions were consistent between the two groups, we observed intriguing associations with several clinicopathological features.

For example, high MARCHF9 expression was significantly associated with advanced disease characteristics. Patients with high MARCHF9 expression exhibited a higher prevalence of distant metastases (pathologic *M* stage) compared to those with low MARCHF9 expression (*P*=0.007). Furthermore, elevated MARCHF9 expression correlated with advanced pathologic stages, with a significantly larger proportion of patients diagnosed at Stage IV in the high MARCHF9 expression group compared to the low expression group (*P*=0.034). Additionally, our analysis unveiled a significant association between elevated MARCHF9 expression and the presence of residual tumors (R1-R2 resection) (*P*=0.010). This finding indicates a potential link between high MARCHF9 expression and more aggressive disease phenotypes, underscoring its clinical relevance in CRC, particularly in advanced stages.

### 3.3. Prognostic Role of MARCHF9 in Colorectal Cancer Survival

**Figure 2:**
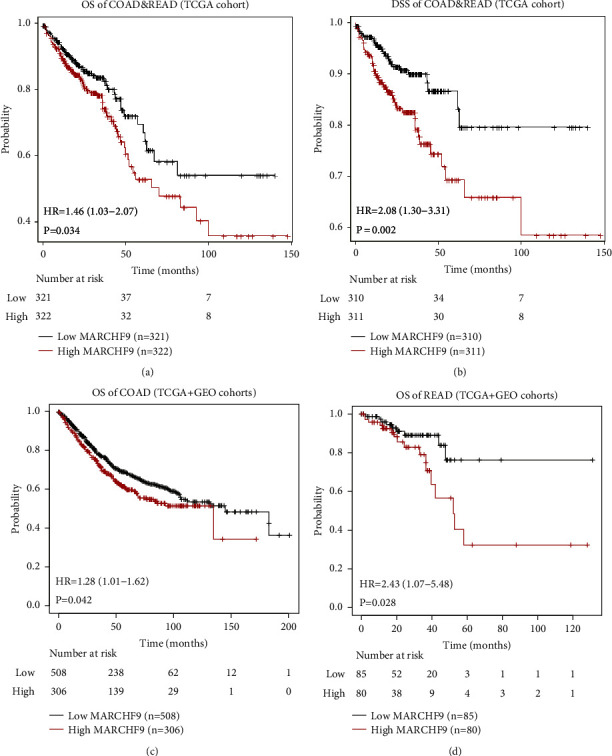
Kaplan–Meier survival curves to assess the prognostic role of MARCHF9. (a) Overall survival (OS) analysis and hazard ratio (HR) of COAD and READ cases in the TCGA cohort according to MARCHF9 expression levels. (b) Disease-specific survival (DSS) analysis and hazard ratio (HR) of COAD and READ cases in the TCGA cohort according to MARCHF9 expression levels. (c) Overall survival analysis and hazard ratio (HR) of COAD cases in a combined cohort of TCGA and GEO datasets, stratified by MARCHF9 expression levels. (d) Overall survival analysis and hazard ratio (HR) of READ cases in a combined cohort of TCGA and GEO datasets, stratified by MARCHF9 expression levels.

To evaluate the prognostic significance of MARCHF9 expression in CRC, we conducted Kaplan–Meier survival analyses using data from both the Cancer Genome Atlas (TCGA) cohort and a mixed cohort that combined TCGA and Gene Expression Omnibus (GEO) data (GSE12945, GSE13294, GSE14333, GSE143985, GSE17538, GSE18088, GSE26682, GSE30540, GSE31595, GSE33114, GSE34489, GSE37892, GSE38832, GSE39582, GSE41258, and GSE92921) [14].  Figure 2 presents the Kaplan–Meier survival curves for various survival endpoints, providing valuable insights into the impact of MARCHF9 expression on patient outcomes.

The overall survival analysis of COAD and READ cases from the TCGA cohort revealed intriguing findings. Patients with high MARCHF9 expression displayed significantly poorer overall survival compared to those with low MARCHF9 expression (Figure 2(a), *P* < 0.05). To further dissect the impact of MARCHF9 on patient outcomes, we assessed disease-specific survival. Similar to overall survival, high MARCHF9 expression was associated with a significantly worse disease-specific survival outcome for COAD and READ cases in the TCGA cohort (Figure 2(b), *P* < 0.05), strengthening the evidence for its prognostic significance in CRC. Expanding our analysis to a mixed cohort that included both TCGA and GEO data, we evaluated the overall survival of COAD cases (Figure 2(c)) and READ cases (Figure 2(d)) [15]. These analyses further supported the notion that elevated MARCHF9 expression is associated with diminished overall survival in both COAD and READ patients, reinforcing its potential as a prognostic marker for CRC across different datasets (*P* < 0.05).

To assess the prognostic significance of MARCHF9 expression and other clinicopathological factors in CRC, we conducted a comprehensive disease-specific survival analysis (Table 2). In our univariate analysis, gender did not demonstrate a significant association with disease-specific survival. Both female and male patients exhibited comparable hazard ratios, with no statistically significant differences observed (*P*=0.412). Age, another potential prognostic factor, also did not show a significant impact on disease-specific survival in the univariate analysis (*P*=0.137). Patients aged 65 years or older displayed a hazard ratio of 1.421 (95% CI: 0.894–2.257) compared to younger patients.

Histological type, distinguishing between adenocarcinoma and mucinous adenocarcinoma, did not reveal a significant difference in disease-specific survival (*P*=0.585). CEA (carcinoembryonic antigen) levels above 5 ng/mL were associated with a significantly increased hazard ratio for disease-specific survival (HR = 2.812, 95% CI: 1.566–5.050, *P* < 0.001). However, in the multivariate analysis, CEA lost its significance as an independent prognostic factor (*P*=0.354). The anatomical neoplasm subdivision (colon vs. rectum) did not exhibit a significant impact on disease-specific survival (*P*=0.779). Pathologic stage emerged as a crucial prognostic factor. In the univariate analysis, advanced stages (TNM Stage III and Stage IV) were associated with significantly higher hazard ratios compared to Stage I (*P* < 0.001). Specifically, Stage IV CRC had a substantial hazard ratio of 27.388 (95% CI: 6.598-113.686). The presence of residual tumors (R1-R2 resection) significantly impacted disease-specific survival, with a hazard ratio of 6.452 (95% CI: 3.789-10.987, *P* < 0.001) in the univariate analysis. In the multivariate analysis, this factor showed a trend towards significance (*P*=0.086).

Crucially, MARCHF9 expression emerged as a robust and independent prognostic factor in both univariate and multivariate analyses. High MARCHF9 expression was associated with a significantly increased hazard ratio for disease-specific survival (HR = 2.077, 95% CI: 1.302–3.312, *P*=0.002) in the univariate analysis, and this significance persisted in the multivariate analysis (HR = 2.510, 95% CI: 1.171–5.382, *P*=0.018). These findings underline the prognostic relevance of MARCHF9 in CRC, suggesting its potential as a valuable biomarker for predicting disease-specific survival outcomes in CRC patients.

### 3.4. Subgroup Overall Survival Analyses of COAD Cases according to MARCHF9 Expression

**Figure 3:**
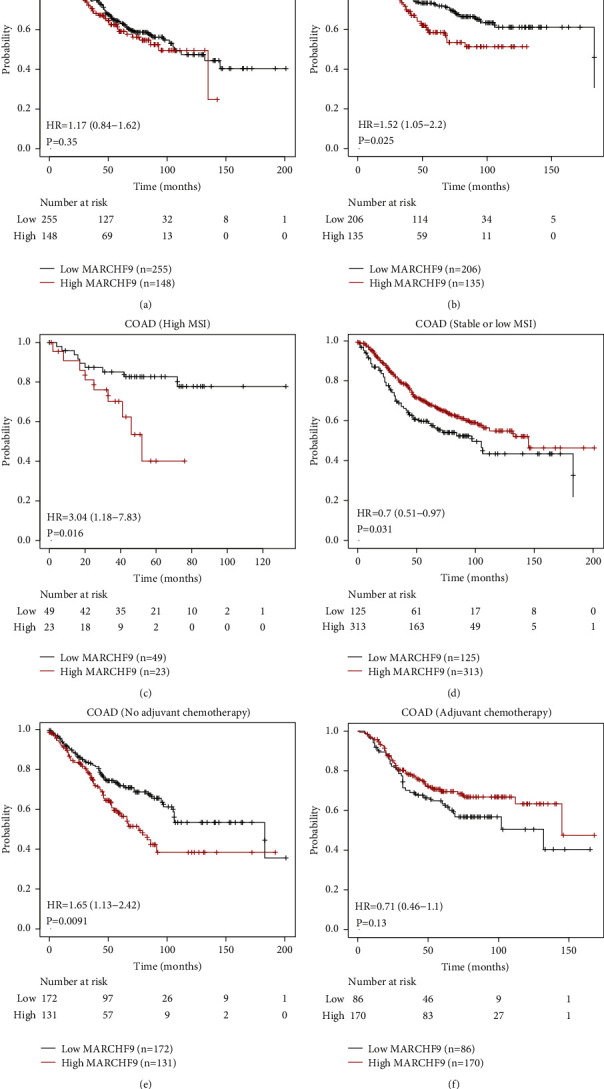
Subgroup overall survival analyses of COAD cases according to MARCHF9 levels. (a, b) Female patients do not exhibit statistically significant differences in survival between the high-MARCHF9 and low-MARCHF9 groups, while male patients display significantly worse survival in the high-MARCHF9 group. (c, d) Elevated MARCHF9 levels predict worse survival in COAD cases with high microsatellite instability (MSI), whereas the opposite trend is observed in COAD cases with low or stable MSI. (e, f) In COAD patients who underwent postoperative adjuvant chemotherapy, higher MARCHF9 levels are correlated with worse prognosis, while no statistically significant association is observed in those who did not receive postoperative adjuvant chemotherapy.

To gain a more nuanced understanding of the prognostic role of MARCHF9 expression in COAD, we conducted subgroup survival analyses (Figure 3). These analyses provide insights into how MARCHF9 expression impacts overall survival within specific subgroups of COAD patients.

For example, we explored the influence of MARCHF9 expression on survival within gender subgroups. Interestingly, female patients displayed no statistical survival difference between the high and low MARCHF9 expression subgroups, suggesting that MARCHF9 may not significantly affect the overall survival of female COAD patients (Figure 3(a), *P*=0.35). In contrast, male patients exhibited a notable difference in survival. Specifically, male patients in the high-MARCHF9 group showed significantly poorer survival outcomes compared to those in the low-MARCHF9 group (Figure 3(b), *P*=0.025). This gender-specific divergence underscores the potential importance of considering gender-related differences in the prognostic impact of MARCHF9 expression in COAD.

Further analyses delve into the relationship between MARCHF9 expression and survival in COAD cases stratified by microsatellite instability (MSI) status. Intriguingly, higher MARCHF9 expression predicted worse survival in COAD patients with high MSI, suggesting that MARCHF9 may have a detrimental impact on prognosis in this specific molecular subgroup (Figure 3(c), *P*=0.016). Conversely, in COAD cases with low or stable MSI, MARCHF9 expression exhibited an opposite significance, potentially indicating a protective or neutral role in survival outcomes (Figure 3(d), *P*=0.031).

Moreover, we assessed the interaction between MARCHF9 expression and postoperative adjuvant chemotherapy in COAD patients. In those who received postoperative adjuvant chemotherapy, higher MARCHF9 expression was correlated with worse prognosis, suggesting that MARCHF9 may negatively impact survival in the context of chemotherapy (Figure 3(e), *P*=0.009). Conversely, in COAD patients who did not receive postoperative adjuvant chemotherapy, MARCHF9 expression showed no statistical significance in predicting survival outcomes (Figure 3(f), *P*=0.13). These findings emphasize the potential relevance of MARCHF9 as a predictive biomarker for treatment response and highlight the need to consider treatment modalities when assessing its prognostic role in COAD.

In summary, these subgroup survival analyses provide valuable insights into the multifaceted prognostic role of MARCHF9 in COAD. The gender-specific differences, MSI-dependent effects, and interactions with adjuvant chemotherapy underscore the complexity of MARCHF9's impact on survival outcomes in COAD patients, highlighting the need for personalized approaches in prognostic assessment and therapeutic decision making.

### 3.5. Inhibition of COAD Cell Proliferation and Invasion through MARCHF9 Silencing

**Figure 4:**
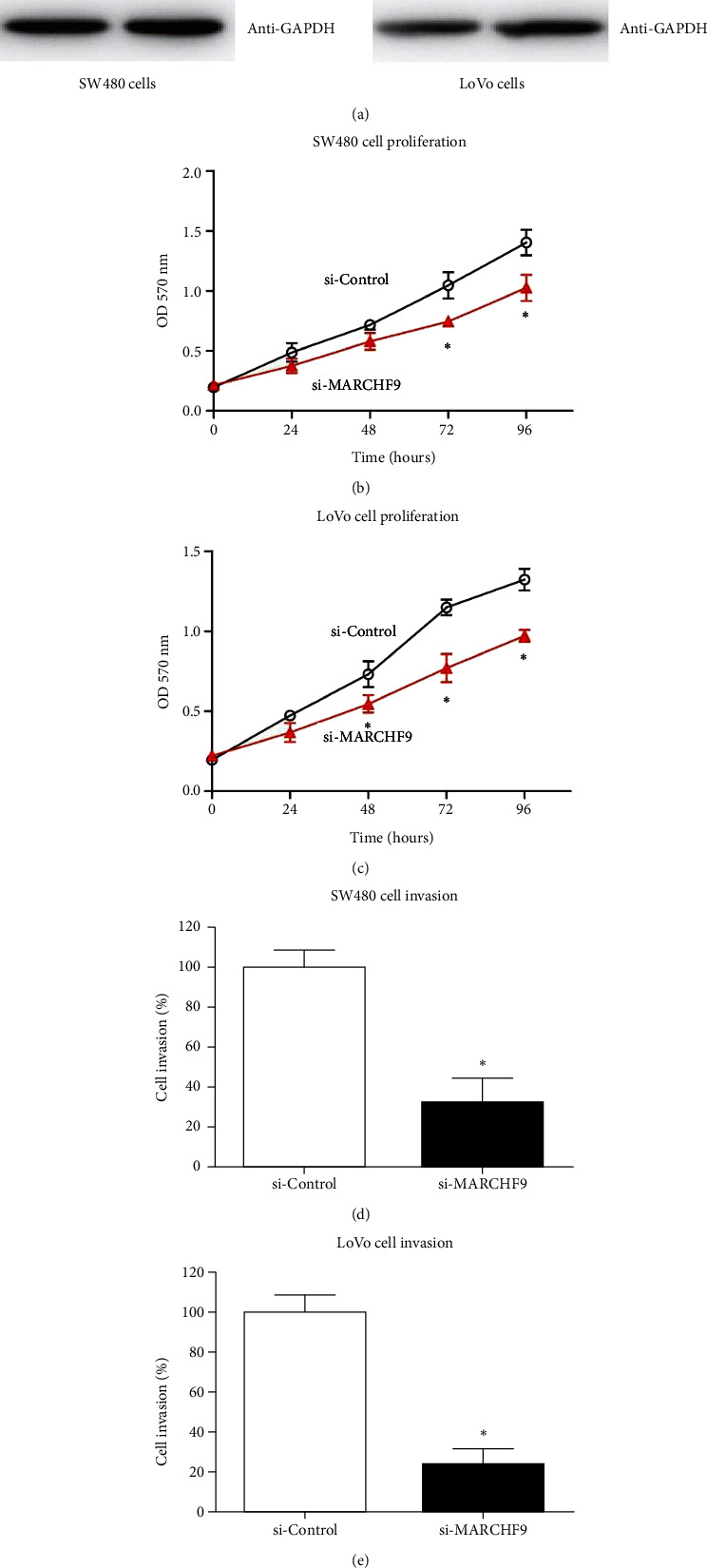
Silencing MARCHF9 inhibits COAD cell proliferation and invasion. (a) Representative western blotting images demonstrating successful knockdown of MARCHF9 in both SW480 and LoVo cell lines. (b, c) Results of the 3-(4,5-dimethylthiazol-2-yl)-2,5-diphenyltetrazolium bromide (MTT) assay indicating that MARCHF9 knockdown significantly decreases the growth rate of COAD cells. (d, e) Matrigel-Transwell assay data illustrating that MARCHF9 knockdown effectively suppresses the invasion of COAD cells. Each experiment was independently repeated three times, and data were statistically compared using Student's *t*-test. ^*∗*^*P* < 0.05.

To explore the functional implications of MARCHF9 in COAD, we next conducted in vitro experiments aimed at silencing MARCHF9 expression in SW480 and LoVo cell lines. Representative western blotting images demonstrate the successful knockdown of MARCHF9 in both SW480 and LoVo cell lines. This knockdown validation underscores the effectiveness of our experimental approach in modulating MARCHF9 expression (Figure 4(a)).

Next, we assessed the impact of MARCHF9 knockdown on COAD cell proliferation, revealing that MARCHF9 knockdown significantly reduced the growth rate of COAD cells compared to control cells (Figures 4(b) and 4(c)). These findings indicate that MARCHF9 plays a crucial role in promoting the proliferation of COAD cells, highlighting its potential as a key regulator of tumor growth. Furthermore, we investigated the influence of MARCHF9 knockdown on COAD cell invasion using Matrigel-Transwell assays. Accordingly, MARCHF9 knockdown led to a notable suppression of COAD cell invasion compared to control cells. This suggests that MARCHF9 may contribute to the invasive properties of COAD cells, further emphasizing its potential as a critical factor in cancer progression (Figures 4(d) and 4(e)).

Therefore, our in vitro experiments demonstrate that silencing MARCHF9 can effectively inhibit the proliferation and invasion of COAD cells. These findings provide valuable mechanistic insights into the oncogenic role of MARCHF9 in COAD and highlight its potential as a promising therapeutic target for colorectal adenocarcinoma.

## 4. Discussion

Colorectal cancer (CRC) remains a significant global health burden, necessitating comprehensive investigations into the molecular mechanisms that drive its pathogenesis [16]. The present study focused on the oncogenic role of Membrane-Associated Ring-CH-Type Finger 9 (MARCHF9), a RING-Type E3 ubiquitin transferase, in CRC. Our findings shed light on the clinical significance of MARCHF9, its functional impact on CRC progression, and its potential as a prognostic biomarker.

Our analysis of data from the Cancer Genome Atlas (TCGA) cohort revealed a substantial increase in MARCHF9 expression in CRC tissues compared to normal colorectal tissues. This upregulation aligns with findings in other cancer types, suggesting that MARCHF9 may serve as an oncogenic factor in multiple malignancies. Interestingly, our study demonstrated that high MARCHF9 expression was associated with advanced clinical stages, distant metastases, and the presence of residual tumors in CRC patients. These clinicopathological associations suggest that MARCHF9 might play a pivotal role in promoting tumor progression and metastasis. The association between MARCHF9 expression and advanced clinical stages is particularly intriguing. This finding echoes prior studies that have implicated MARCHF9 in cancer progression. Survival analyses in our study demonstrated that high MARCHF9 expression was significantly associated with unfavorable overall and disease-specific survival outcomes in CRC patients. This observation underscores MARCHF9's potential as a prognostic biomarker in CRC, aligning with emerging evidence from other cancer types. Notably, MARCHF9 has been implicated in the prognosis of lung adenocarcinoma, where it suppresses tumor progression by downregulating ICAM-1 [17]. Low MARCHF9 expression has been linked to poor prognosis and adverse clinicopathological characteristics of lung adenocarcinoma. The distinct pattern of MARCHF9's association with poor survival outcomes across various cancers suggests its broad applicability as a prognostic marker. Of note, in our multivariate analysis, the significance of the pathological stage (TNM) was compromised, which may be due to collinearity with other variables. The multivariate model included several interrelated factors that could impact the results, causing the pathological stage to lose its statistical significance (*P*=0.997), emphasizing the need for cautious interpretation of these findings.

In vitro experiments in our study revealed that siRNA-mediated knockdown of MARCHF9 in COAD cell lines (SW480 and LoVo) led to a significant reduction in cell proliferation and invasion. These findings highlight the functional relevance of MARCHF9 in promoting CRC progression. While the exact mechanisms underlying MARCHF9's oncogenic effects in CRC warrant further investigation, it is conceivable that MARCHF9 may impact key pathways involved in cell proliferation, invasion, and metastasis. The role of MARCHF9 in promoting cancer cell proliferation has been documented in other malignancies. In glioblastoma, MARCHF9 has been implicated in tumor immune microenvironment [18]. Our findings align with these reports and suggest that MARCHF9's pro-proliferative effects may extend to CRC. Moreover, our study demonstrated that MARCHF9 knockdown significantly inhibited the invasion of COAD cells. The inhibition of COAD cell invasion upon MARCHF9 knockdown in our study suggests that MARCHF9 may similarly influence the invasive behavior of CRC cells.

However, it is essential to acknowledge differences in MARCHF9's role across cancer types. For instance, in lung adenocarcinoma, MARCHF9 overexpression has been correlated with favorable clinicopathological characteristics and can inhibit tumor invasion while showing little effect on cell proliferation [17]. In glioblastoma, MARCHF9 has been reported to be involved in suppressive immune microenvironments [18]. In summary, while MARCHF9's clinical significance is widely recognized, its functional roles and mechanisms of action may vary depending on the specific cancer type.

The clinical significance of MARCHF9 in CRC, as highlighted in our study, presents opportunities for its translation into clinical practice. MARCHF9's association with advanced disease stages, distant metastases, and poor prognosis suggests its potential utility as a prognostic biomarker. Patients with high MARCHF9 expression may benefit from more intensive monitoring and personalized treatment strategies. Moreover, our functional findings suggest that MARCHF9 may serve as a promising therapeutic target in CRC. Strategies aimed at inhibiting MARCHF9 expression or activity could be explored to impede cancer cell proliferation and invasion. Given the heterogeneity of CRC, patient stratification based on MARCHF9 expression levels may help identify individuals who are most likely to respond to targeted therapies.

While our analysis was based on substantial datasets from TCGA and GEO, retrospective analyses inherently have limitations related to data quality and potential confounders. Further prospective studies and functional experiments are warranted to validate our findings and elucidate the exact mechanisms underlying MARCHF9's oncogenic effects in CRC. In addition, future research endeavors should delve into elucidating the precise molecular mechanisms by which MARCHF9 influences CRC progression. Investigating its downstream targets and interacting partners could provide valuable insights into potential therapeutic interventions. Additionally, the impact of MARCHF9 in preclinical models and its evaluation as a therapeutic target in clinical trials should be explored to assess its translational potential.

## 5. Conclusions

In conclusion, our study illuminates the oncogenic role of MARCHF9 in colorectal cancer. High MARCHF9 expression is associated with advanced disease stages, metastasis, and adverse prognosis, while in vitro experiments demonstrate its functional impact on cancer cell proliferation and invasion. These findings underscore MARCHF9's clinical relevance as a potential prognostic biomarker and therapeutic target in CRC.

## Figures and Tables

**Table 1:** Clinical characteristics of colorectal cancer patients stratified by MARCHF9 expression.

Characteristics	Low expression of MARCHF9	High expression of MARCHF9	*P* value
Total cases, *n*	322	322	
Gender, *n* (%)			0.693
Female	153 (50.8%)	148 (49.2%)	
Male	169 (49.3%)	174 (50.7%)	
Age, *n* (%)			0.524
≤65 yrs	142 (51.4%)	134 (48.6%)	
>65 yrs	180 (48.9%)	188 (51.1%)	
Pathologic *T* stage, *n* (%)			0.205
T1	12 (60%)	8 (40%)	
T2	50 (45%)	61 (55%)	
T3	227 (52.1%)	209 (47.9%)	
T4	31 (41.9%)	43 (58.1%)	
Pathologic *N* stage, *n* (%)			0.404
N0	190 (51.6%)	178 (48.4%)	
N1	76 (49.7%)	77 (50.3%)	
N2	53 (44.5%)	66 (55.5%)	
Pathologic *M* stage, *n* (%)			0.007
M0	245 (51.6%)	230 (48.4%)	
M1	32 (36%)	57 (64%)	
Pathologic stage, *n* (%)			0.034
Stage I	52 (46.8%)	59 (53.2%)	
Stage II	129 (54.2%)	109 (45.8%)	
Stage III	95 (51.6%)	89 (48.4%)	
Stage IV	33 (36.7%)	57 (63.3%)	
Histological type, *n* (%)			0.525
Adenocarcinoma	271 (49.3%)	279 (50.7%)	
Mucinous adenocarcinoma	44 (53%)	39 (47%)	
Residual tumor, *n* (%)			0.010
R0	231 (49.4%)	237 (50.6%)	
R1-R2	12 (28.6%)	30 (71.4%)	
CEA level, *n* (%)			0.850
≤5 ng/mL	133 (51%)	128 (49%)	
>5 ng/mL	77 (50%)	77 (50%)	
Anatomic neoplasm subdivision, *n* (%)			0.411
Colon^#^	256 (50.7%)	249 (49.3%)	
Rectum	51 (46.4%)	59 (53.6%)	

^#^Ascending colon (89 cases), cecum (112 cases), descending colon (20 cases), hepatic flexure (26 cases), rectosigmoid junction (5 cases), sigmoid colon (166 cases), splenic flexure (7 cases), and transverse colon (40 cases).

**Table 2:** Disease-specific survival of colorectal cancer patients.

Characteristics	Total (*N*)	Univariate analysis		Multivariate analysis	
		Hazard ratio (95% CI)	*P* value	Hazard ratio (95% CI)	*P* value
Gender	621				
Female	290	Reference			
Male	331	1.207 (0.769–1.895)	0.412		
Age	621				
≤65 yrs	273	Reference			
>65 yrs	348	1.421 (0.894–2.257)	0.137		
Histological type	612				
Adenocarcinoma	533	Reference			
Mucinous adenocarcinoma	79	1.195 (0.631–2.262)	0.585		
CEA level	413				
≤5 ng/mL	259	Reference		Reference	
>5 ng/mL	154	2.812 (1.566–5.050)	<0.001	1.393 (0.691–2.810)	0.354
Anatomic neoplasm subdivision	594				
Colon	487	Reference			
Rectum	107	0.912 (0.481–1.732)	0.779		
Pathologic stage	601				
Stage I	111	Reference		Reference	
Stage II	228	2.741 (0.618–12.165)	0.185	69587464.4550 (0.000-Inf)	0.997
Stage III	174	6.688 (1.571–28.477)	0.010	98248212.7528 (0.000-Inf)	0.997
Stage IV	88	27.388 (6.598–113.686)	<0.001	325344207.7492 (0.000-Inf)	0.997
Residual tumor	508				
R0	466	Reference		Reference	
R1-R2	42	6.452 (3.789–10.987)	<0.001	2.023 (0.904–4.525)	0.086
MARCHF9	621				
Low	310	Reference		Reference	
High	311	2.077 (1.302–3.312)	0.002	2.510 (1.171–5.382)	0.018

## Data Availability

Data will be available upon reasonable request.

## References

[1] Ferlay J., Colombet M., Soerjomataram I. (2019). Estimating the global cancer incidence and mortality in 2018: GLOBOCAN sources and methods. *International Journal of Cancer*.

[2] Labianca R., Beretta G. D., Kildani B. (2010). Colon cancer. *Critical Reviews in Oncology/Hematology*.

[3] Jung G., Hernández-Illán E., Moreira L., Balaguer F., Goel A. (2020). Epigenetics of colorectal cancer: biomarker and therapeutic potential. *Nature reviews Gastroenterology and Hepatology*.

[4] Sun J., Dong Z., Chang Z. (2021). MARCH6 promotes hepatocellular carcinoma development through up-regulation of ATF2. *BMC Cancer*.

[5] Senft D., Qi J., Ronai Z. A. (2018). Ubiquitin ligases in oncogenic transformation and cancer therapy. *Nature Reviews Cancer*.

[6] de Angelis Rigotti F., De Gassart A., Pforr C. (2017). MARCH9‐mediated ubiquitination regulates MHC I export from the TGN. *Immunology and Cell Biology*.

[7] Lin M., Jin Y., Wang F. (2023). MARCH9 mediates NOX2 ubiquitination to alleviate NLRP3 inflammasome-dependent pancreatic cell pyroptosis in acute pancreatitis. *Pancreas*.

[8] Tan C., Byrne E. F. X., Ah-Cann C., Call M. J., Call M. E. (2019). A serine in the first transmembrane domain of the human E3 ubiquitin ligase MARCH9 is critical for down-regulation of its protein substrates. *Journal of Biological Chemistry*.

[9] Mao G., Zhou B., Xu W. (2021). Hsa_circ_0040809 regulates colorectal cancer development by upregulating methyltransferase DNMT1 via targeting miR‐515‐5p. *The Journal of Gene Medicine*.

[10] Liu H., Chen B., Liu L. L., Cong L., Cheng Y. (2022). The role of MARCH9 in colorectal cancer progression. *Frontiers in Oncology*.

[11] Zhou M., He S. J., Liu W/ (2022). EZH2 upregulates the expression of MAPK1 to promote intervertebral disc degeneration via suppression of miR‐129‐5p. *The Journal of Gene Medicine*.

[12] Xu K., Li S., Yang Q. (2021). MicroRNA‐145‐5p targeting of TRIM2 mediates the apoptosis of retinal ganglion cells via the PI3K/AKT signaling pathway in glaucoma. *The Journal of Gene Medicine*.

[13] Zhang C., Liu Z., Sheng Y. (2022). PRDM5 suppresses oesophageal squamous carcinoma cells and modulates 14–3‐3zeta/Akt signalling pathway. *Clinical and Experimental Pharmacology and Physiology*.

[14] Kovács S. A., Fekete J. T., Győrffy B. (2023). Predictive biomarkers of immunotherapy response with pharmacological applications in solid tumors. *Acta Pharmacologica Sinica*.

[15] Nagy Á., Munkácsy G., Győrffy B. (2021). Pancancer survival analysis of cancer hallmark genes. *Scientific reports*.

[16] Sveen A., Kopetz S., Lothe R. A. (2020). Biomarker-guided therapy for colorectal cancer: strength in complexity. *Nature Reviews Clinical Oncology*.

[17] Shen Q. M., Wang H. Y., Xu S. (2018). MARCH9 suppresses lung adenocarcinoma progression by downregulating ICAM-1. *Cellular Physiology and Biochemistry*.

[18] Luoto S., Hermelo I., Vuorinen E. M. (2018). Computational characterization of suppressive immune microenvironments in glioblastoma. *Cancer Research*.

